# Na_3_MnTi(PO_4_)_3_/C Nanofiber Free-Standing Electrode for Long-Cycling-Life Sodium-Ion Batteries

**DOI:** 10.3390/nano14090804

**Published:** 2024-05-05

**Authors:** Debora Maria Conti, Claudia Urru, Giovanna Bruni, Pietro Galinetto, Benedetta Albini, Vittorio Berbenni, Alessandro Girella, Doretta Capsoni

**Affiliations:** 1 Department of Chemistry, Physical Chemistry Section & C.S.G.I. (Consorzio Interuniversitario per lo Sviluppo dei Sistemi a Grande Interfase), University of Pavia, Via Taramelli 16, 27100 Pavia, Italy; deboramaria.conti01@universitadipavia.it (D.M.C.); claudia.urru01@universitadipavia.it (C.U.); giovanna.bruni@unipv.it (G.B.); vittorio.berbenni@unipv.it (V.B.); alessandro.girella@unipv.it (A.G.); 2 Department of Physics, University of Pavia, Via Bassi 6, 27100 Pavia, Italy; pietro.galinetto@unipv.it (P.G.); benedetta.albini@unipv.it (B.A.)

**Keywords:** Na-ion batteries, Na_3_MnTi(PO_4_)_3_, NASICON-type electrode, self-standing electrode, carbon nanofibers

## Abstract

Self-standing Na_3_MnTi(PO_4_)_3_/carbon nanofiber (CNF) electrodes are successfully synthesized by electrospinning. A pre-synthesized Na_3_MnTi(PO_4_)_3_ is dispersed in a polymeric solution, and the electrospun product is heat-treated at 750 °C in nitrogen flow to obtain active material/CNF electrodes. The active material loading is 10 wt%. SEM, TEM, and EDS analyses demonstrate that the Na_3_MnTi(PO_4_)_3_ particles are homogeneously spread into and within CNFs. The loaded Na_3_MnTi(PO_4_)_3_ displays the NASICON structure; compared to the pre-synthesized material, the higher sintering temperature (750 °C) used to obtain conductive CNFs leads to cell shrinkage along the *a* axis. The electrochemical performances are appealing compared to a tape-casted electrode appositely prepared. The self-standing electrode displays an initial discharge capacity of 124.38 mAh/g at 0.05C, completely recovered after cycling at an increasing C-rate and a coulombic efficiency ≥98%. The capacity value at 20C is 77.60 mAh/g, and the self-standing electrode exhibits good cycling performance and a capacity retention of 59.6% after 1000 cycles at 1C. Specific capacities of 33.6, 22.6, and 17.3 mAh/g are obtained by further cycling at 5C, 10C, and 20C, and the initial capacity is completely recovered after 1350 cycles. The promising capacity values and cycling performance are due to the easy electrolyte diffusion and contact with the active material, offered by the porous nature of non-woven nanofibers.

## 1. Introduction

The increasing world energy demand and the need to face global climate changes and environmental pollution concerns urgently call for the development of renewable energy sources such as solar, water, and wind [1,2,3]. Rechargeable batteries play a relevant role in large-scale renewable and clean energy storage [4,5,6]. Despite the fact that lithium-ion batteries (LIBs) exhibit a high operating voltage, high energy density, and improved specific capacity and lifespan [7,8,9,10,11] and represent a mature and performant technology in energy storage systems, the scarce and uneven distribution of lithium resources, as well as the rising cost, make them not suitable to fulfil large-scale applications [5,12,13,14]. Sodium-ion batteries (SIBs) represent a fascinating candidate for next-generation large-scale grid energy systems: SIBs’ working mechanism is comparable to LIBs’. Sodium is abundant on the Earth’s crust and seawater, and it is inexpensive [15,16,17]. Compared to lithium, sodium displays similar chemical, electrochemical, and physical properties, but its larger cation size (1.02 Å) and slower diffusion rate cause huge volume expansions during its insertion into the electrode material. This feature poses concerns on the SIBs’ requisites such as adequate values of specific capacities, a sufficient coulombic efficiency, and long lifespan. The cathode materials play a relevant role in SIBs, and numerous compounds displaying open framework structures and suitable tunnels for Na-ion diffusion have been investigated, such as Prussian blue analogues [18,19,20,21,22,23], O3- and P2-type transition metal layered oxides [24,25,26,27,28], and polyanionic compounds [29,30,31,32,33,34]. Among the polyanionic phosphates, Na superionic conductor (NASICON)-structured compounds are appealing candidates as cathodes for SIBs. Na_3_V_2_(PO_4_)_3_ is considered the most representative: the Na ions partially occupy two crystallographic sites, and cation transport is assured by the open three-dimensional structure based on corner-sharing VO_6_ octahedra and PO_4_ tetrahedra to form V_2_(PO_4_)_3_ repeating units [35]. More recently, research has focused on NASICON-structured mixed transition metal phosphates, suitable to access high voltages. Among them, Na_3_MnTi(PO_4_)_3_ seems very promising. Gao and co-workers [36,37] detected two high-voltage plateaus at about 3.6 and 4.1 V, attributed to the Mn^3+^/Mn^2+^ and Mn^4+^/Mn^3+^ redox processes (the extraction of two sodium ions). After cycling and repeated intercalation/deintercalation processes, small volume changes and structural stability were detected. The Zhu research group [38] synthesized, through the spray-drying-assisted route, Na_3_MnTi(PO_4_)_3_/C hollow microsphere cathodes, which exhibited a fully reversible three-sodium-ion extraction/insertion. The peaks at about 2.1, 3.5 and 4.0 V vs. Na^+^/Na correspond to the Ti^3+^/Ti^4+^, Mn^2+^/Mn^3+^, and Mn^3+^/Mn^4+^ redox reactions. A high specific capacity of 160 mAh/g at 0.2 C is achieved, comparable to the theoretical one (176 mAh/g). Despite the intriguing electrochemical properties of the Na_3_MnTi(PO_4_)_3_ cathode, its poor electronic conductivity and the presence of intrinsic anti-site defects involving the Mn occupation of vacancies on Na(2) site cause voltage hysteresis and inhibit good cycling stability and rate performance [39]. Several strategies were investigated to overcome these problems: carbon coating, the synthesis of tailored morphologies/nanostructures [40,41], and the doping/partial substitution of transition metal ions [39,42,43,44,45].

In the last decade, carbon nanofibers (CNFs) proved to be appealing materials for electrochemical applications. The good electronic conductivity and mechanical properties make them suitable conductive fillers or conductive supports of electrode active materials [46,47,48,49,50,51]. Noteworthy, the CNFs themselves are promising anodes [52]: CNFs display a high surface area and electronic conduction thanks to their unique 1D morphology. They can be obtained by electrospinning, a simple and scalable technology: a polymeric solution is electrospun, stabilized at about 250 °C in air, and carbonized at 800–900 °C in an inert atmosphere. The obtained non-woven CNFs display good electronic conductivity and desirable porosity to allow electrolyte permeation and to buffer the volume changes occurring with sodiation/desodiation. Notably, the mechanical properties of CNFs make them suitable to fabricate self-standing electrodes, avoiding the use of the metal current collector. Two approaches are mainly used to synthesize active materials/CNFs self-standing electrodes, both viable and flexible. In the first method [46,47,48], a solution containing active material precursors and a polymer is electrospun and thermal-treated at temperatures suitable to carbonize nanofibers and synthesize the active material. Via the second route [50,51], the solution containing active material precursors is dip-/drop-coated on CNFs obtained by electrospinning, and the fibers are thermal-treated at the synthesis temperature of the active material. In both approaches the active material is synthesized in situ, but some limits can be envisaged: (i) the temperature/atmosphere of the active materials synthesis and nanofibers carbonization mismatching and (ii) the non-homogeneous distribution of the active material along the fibers’ thickness by dip/drop-coating.

In this paper, we propose a different approach to synthesize Na_3_MnTi(PO_4_)_3_/CNF composites by electrospinning. The active material, synthesized ex situ by the sol–gel route, is added in proper amounts (10 and 30 wt%) to a polyacrylonitrile in N, N-dimethylacetamide solution; the dispersion is electrospun, and the sheets are stabilized and carbonized to obtain self-standing electrodes. The composites are characterized by different techniques (X-ray powder diffraction and Rietveld structural and profile refinement, scanning electron microscopy, energy dispersive spectroscopy, transmission electron microscopy, Raman spectroscopy, and thermogravimetry) to investigate the structure and morphology of and active material distribution in CNFs. The electrochemical performances of the Na_3_MnTi(PO_4_)_3_/CNF electrodes are tested and discussed, compared to a Na_3_MnTi(PO_4_)_3_ tape-casted electrode (70 wt% active material). An improved capacity at high C-rates and cell lifespan are achieved, thanks to the benefits of CNFs: good electronic conductivity, easy electrolyte permeation into the self-standing electrode, and porous fibers’ ability to buffer the active material volume change during sodiation/desodiation.

## 2. Materials and Methods

### 2.1. Materials

Sodium acetate (CH_3_COONa; Aldrich, Milan, Italy, 99%), manganese (II) acetate tetrahydrate (CH_3_COO)_2_Mn·4H_2_O; Aldrich, Milan, Italy, 99%), ammonium phosphate monobasic (NH_4_H_2_PO_4_; Aldrich, Milan, Italy, 99%), titanium (IV) isopropoxide (C_12_H_28_O_4_Ti; Aldrich, Milan, Italy, 99%), absolute ethanol (C_2_H_5_OH, Aldrich, Milan, Italy), citric acid (C_6_H_8_O_7_; Aldrich, Milan, Italy, 99%), polyacrylonitrile (PAN: (C_3_H_3_N)_n_; Aldrich, Milan, Italy, particle size 50 µm, 99.5% AN/0.5% MA), N,N-dimethylacetamide (DMAc: CH_3_CON(CH_3_)_2_; Aldrich, Milan, Italy, 99%), Super P carbon, polyvinylidene fluoride (PVdF Kynar), N-methyl-2-pyrrolidone (NMP: Aldrich, Milan, Italy, 99%), 1M sodium perchlorate (NaClO_4_) in propylene carbonate (PC) 1:1 v:v (Aldrich, Milan, Italy, 98%), and 4-Fluoro-1,3-diocolan-2-one (FEC; Aldrich, Milan, Italy, 99.5%) were employed to synthesize the active material and the self-standing electrodes and to prepare the slurry of the tape-casted electrode and the electrolyte.

### 2.2. Synthesis of Electrode Materials

The Na_3_MnTi(PO_4_)_3_ active material is synthesized via sol–gel [53]. An aqueous solution of CH_3_COONa, (CH_3_COO)_2_Mn·4H_2_O, NH_4_H_2_PO_4_, and citric acid is added dropwise to a solution of titanium (IV) isopropoxide in absolute ethanol. The reagents are taken in a stoichiometric amount, and citric acid equals the transition metal moles. The solution is stirred at 80 °C until a gel forms. The gel is dried at 100 °C, ground in an agate mortar, and heat-treated at 650 °C under nitrogen atmosphere for 12 h. From now on, the powder sample is indicated by the code MnTi.

The self-standing electrodes are synthesized by electrospinning. The electrospun dispersion is prepared by adding MnTi active material (10 and 30 wt%) to a solution of PAN (8 wt%) in DMAc [49,51]. Hereafter, we report the detailed synthesis of the two self-standing electrodes (codes: 10%MnTi/CNF and 30%MnTi/CNF). The MnTi powder is ball-milled at 100 rpm for two cycles (20 min each), then 10 wt% of MnTi (0.376 g) or 30 wt% of MnTi (1.128 g) is added to DMAc (50 mL). The suspension is sonicated for 1 h, after which 3.760 g PAN is added, and the suspension is stirred overnight at 60 °C. The solution is electrospun by using the EF050 Starter Kit Electrospinning system of SKE Research Equipment (C/O Leonardino S.r.l, Bollate, MI, Italy), setting the following conditions: 10.5 mL dispersion, 3.5 mL/h flow, 16 gauge needle, applied voltage 16 kV, needle–collector distance 18 cm, and deposition time 3 h. The setting parameters had been previously optimized. Finally, a homemade humidity sensor-included box is built for humidity control: a value lower than 20% is detected during all depositions.

The same electrospinning procedure is applied to a solution of PAN (8 wt%) in DMAc to prepare a pure CNF sample: it is used for comparison with the self-standing electrode’s characterization.

The electrospun sheets are removed from the support (aluminum foil) and stabilized in air for 30 min at 100 °C, 30 min at 200 °C, and finally 2 h at 260 °C (heating ramp: 5 °C min^−1^). They are further heat-treated at 750 °C for 2 h (heating ramp: 10 °C min^−1^) in nitrogen atmosphere for the carbonization process.

### 2.3. Materials Characterization

X-ray powder diffraction (XRPD) measurements are performed by using a Bruker D5005 diffractometer with the Cu Kα radiation (40 kV, 40 mA) and a scintillation detector. The patterns are collected in the 18–80° 2θ range with a step size of 0.03° and 22 s/step counting time. The Rietveld structural refinement is applied to the diffraction data. The NASICON-type structure model (S.G. $R\bar{3}c$) is used to determine the main structural parameters. The TOPAS V3.0 software is used [54].

SEM micrographs are collected by a Zeiss EVO MAH10 (Carl Zeiss, Oberkochen, Germany) scanning electron microscope on Au-sputtered samples (20 kV, secondary electron images, working distance 8.5 mm). The microscope is equipped with an energy dispersive detector (X-max 50 mm^2^, Oxford Instruments, Oxford, UK) for the EDS analysis.

TEM images are collected on JEOL JEM-1200EXIII equipped with a TEM CCD camera Mega View III transmission electron microscope to highlight the presence of MnTi powder in the CNFs.

The TGA data collection is performed with a TA Q5000 instrument in air in the 20–725 °C temperature range (heating rate: 10 Kmin^−1^). The technique is used to determine the effective weight percentage of MnTi powder in CNFs.

The Raman measurements are performed employing a microRaman spectrometer, XploRA Plus HORIBA Scientific (Kyoto, Japan), equipped with an Olympus BX43 microscope. Laser red light at 638 nm (90 mW) is used as the excitation source. The incident laser power is tuned by a set of neutral filters with different optical densities. The investigated samples are placed on a motorized xy stage. The spectral resolution is about 2 cm^−1^. An open electrode CCD camera, with a multistage Peltier air-cooling system, is used as a detector. The measurements are performed using a 50× objective with a long working distance, which leads to a spatial resolution of the order of 4 μm. The spectra have been collected with a mean integration time of about 10 s and a number of accumulations equal to 10. All the reported data are the result of the average of different spectra collected at different points in each sample.

### 2.4. Electrochemical Characterization

A Swagelok cell is used for the electrochemical investigation. The cells are assembled in an argon-filled dry box (M. Braun H_2_O < 0.1 ppm; O_2_ < 0.1 ppm) by using the self-standing electrodes (see Section 2.2), 1 M NaClO_4_ in PC and 5% FEC as the electrolyte, and sodium foil as the counter-electrode.

For comparison, a Swagelok cell is assembled by using a tape-casted electrode: to prepare the Na_3_MnTi(PO_4_)_3_ slurry, the MnTi powder is ball-milled at 100 rpm for two cycles (20 min each). A mixture of 70 wt% active material, 20 wt% Super P carbon and 10 wt% PVdF is stirred in NMP for 2 h, cast on aluminum foil, and dried at 70 °C for 3 h.

The electrochemical properties are investigated at ambient temperature by cyclic voltammetry (CV) and galvanostatic charge/discharge cycles. The CV is performed with an Autolab potentiostat. All cells are cycled in the 1.5–4.5 V potential range. Galvanostatic charge/discharge cycles are obtained with a Neware-4000BTS apparatus at different current rates in the abovementioned potential range. The electrochemical impedance spectroscopy (EIS) measurements were performed on an Autolab PGSTAT30 potentiostat (Eco Chemie). The EIS spectra were acquired at OCV in the 10^5^–10^−2^ Hz frequency range with an amplitude potential of 1 mV.

## 3. Results and Discussion

### 3.1. Structural Characterization

**Figure 1:**
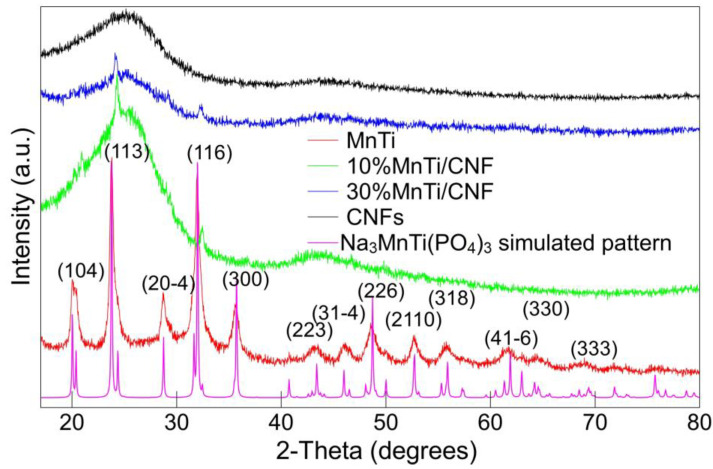
X-ray diffraction patterns of the MnTi, MnTi/CNF, and pure CNF samples. The Na_3_MnTi(PO_4_)_3_ simulated pattern is also shown.

In Figure 1, the diffraction patterns of all samples are shown. The MnTi sample displays the diffraction peaks of the NASICON-type crystal structure and well compares to the literature data [53]; no impurity phases are detected. The pure CNF sample displays a broad band at about 25°/2θ, typical of amorphous components. In the MnTi/CNF samples, the peaks of the NASICON structure and the amorphous CNF phase are detected. Rietveld refinement is applied to the MnTi, 10%MnTi/CNF and 30%MnTi/CNF diffraction patterns. The structural model reported by Zhou and co-workers [53] is used ($R\bar{3}c$ S.G. and lattice parameters a = 8.73352 Å and c = 21.84703 Å). The lattice parameters, crystallite size, and degree of crystallinity obtained by Rietveld refinement are reported in Table S1 (Supporting Information). Figure S1 (Supporting Information) compares the experimental and calculated patterns.

The discrepancy factor values (Table S1) and the graphical comparison (Figure S1) demonstrate that the refined model properly fits the experimental XRPD data. The refined lattice parameters of the MnTi sample well compare to the literature ones [53]. However, for both MnTi/CNF samples, the *a* lattice parameter and cell volume decrease, giving rise to a *c/a* ratio increase (Table S1). The cell volume shrinkage may depend on the sintering temperature of 750 °C used for the carbonization process and not on the Na_3_MnTi(PO_4_)_3_ inclusion in carbon nanofibers. In fact, the same shrinkage was detected for Na_3_MnTi(PO_4_)_3_ samples synthesized at the same temperature by Liu and coworkers [55]. Moreover, the temperature higher than that usually chosen in the sol–gel synthesis (650 °C) may increase intrinsic anti-sites defects, responsible for the charge/discharge voltage hysteresis [39]. The cell shrinkage inhibits the Na(2) reversible insertion/extraction and impacts the electrochemical performances, as discussed in 3.5. However, we chose the carbonization temperature of 750 °C based on the increased conductivity of pure CNFs: 1.56 × 10^−3^ S/cm and 1.76 × 10^−6^ S/cm for carbonization at 750 °C and 650 °C, respectively. The Na_3_MnTi(PO_4_)_3_ active material in both MnTi and MnTi/CNF samples is nanocrystalline (Table S1), and the crystallite size does not depend on the sintering temperature. The degree of crystallinity gives an idea of the amount of crystalline phase (active material) loaded in the self-standing electrodes. The degree of crystallinity is about 9% for the MnTi/CNF samples (Table S1). The value matches the synthesis content of the 10%MnTi/CNF but not the 30%MnTi/CNF one. It may depend on the possible decantation of MnTi powder into the tube during the electrospinning deposition. The quantitative amount of the active material loaded into CNFs will be evaluated by TGA and discussed in 3.3.

### 3.2. Samples Morphology

**Figure 2:**
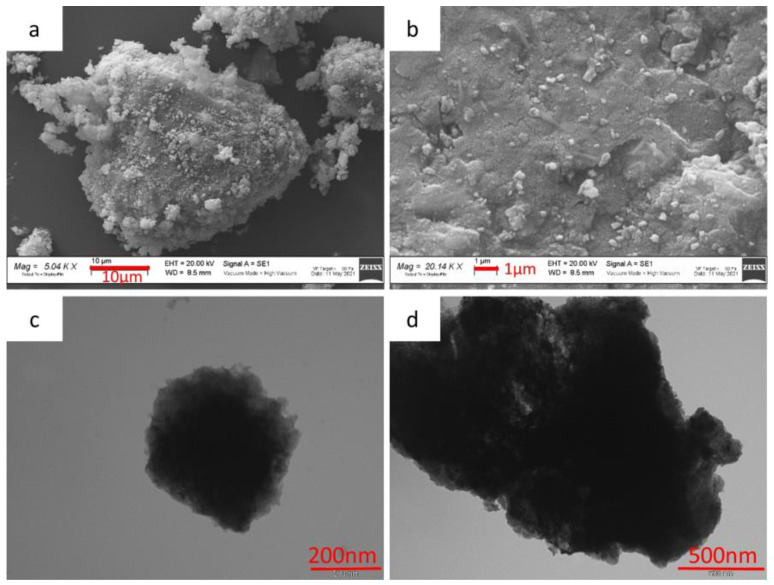
SEM (**a**,**b**) and TEM (**c**,**d**) images of the MnTi sample.

Figure 2a,b shows the SEM images of the MnTi sample. Large aggregates (10–30 µm), composed of sub-particles smaller than 1 µm, are observed. The grain’s surfaces are rough but well defined. The TEM micrographs in Figure 2c,d confirm the presence of the particles’ aggregates. Noteworthy, the particulate is surrounded by homogeneously spread carbon coating due to the carbon source (citric acid) used in the sol–gel synthesis.

**Figure 3:**
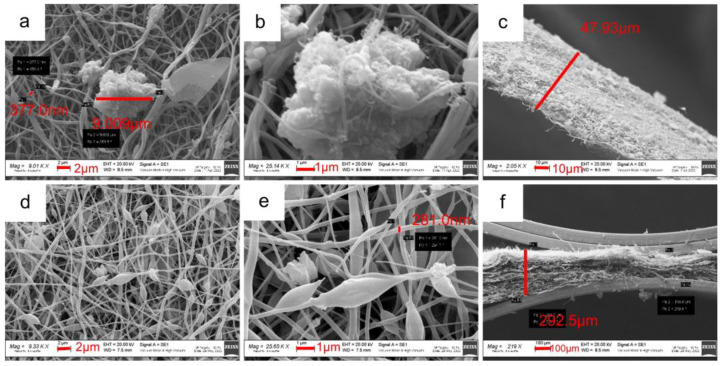
SEM images of surface (**a**,**b**) and cross-section (**c**) of the 10%MnTi/CNF sample. SEM images of surface (**d**,**e**) and cross-section (**f**) of the 30%MnTi/CNF sample.

The SEM surface and cross-section images of the 10%MnTi/CNF sheets (Figure 3a–c) demonstrate that the Na_3_MnTi(PO_4_)_3_ is dispersed into nanofibers and forms agglomerates with a widely varying size distribution. The 10%MnTi/CNF sheet thickness is about 50 μm. As in the case of the 10%MnTi/CNF, the SEM surface and cross-section images of the 30%MnTi/CNF sample (Figure 3d–f) display agglomerates with an uneven size spread in the CNF matrix. The sheet thickness is about 300 µm. The carbonization process does not influence the homogeneous distribution of the active material, as demonstrated by the SEM images taken on the electrospun and graphitized 10%MnTi/CNF sample (Figure S2). As expected, the carbonization process causes a slight decrease in CNFs’ diameter.

**Figure 4:**
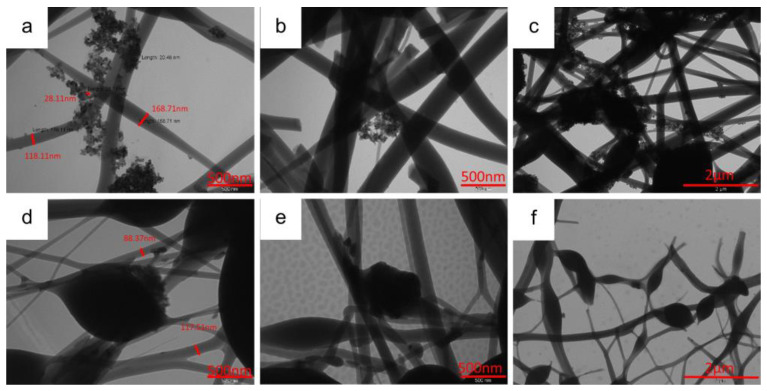
TEM images of 10%MnTi/CNF (**a**–**c**) and 30%MnTi/CNF (**d**–**f**) samples taken at different magnifications.

The morphology, particle size, and distribution of the active material in self-standing electrodes are deeply investigated by TEM analysis. The TEM images of the 10%MnTi/CNF sample (Figure 4a–c) display Na_3_MnTi(PO_4_)_3_ nanoparticles of about 20–30 nm, consistent with the crystallite size reported in Table S1; they form aggregates of variable size and segregate between and into nanofibers (CNFs diameter: 120–170 nm). The 30%MnTi/CNF sample displays comparable morphology (Figure 4d–f), and the Na_3_MnTi(PO_4_)_3_ aggregates are spread both between and embedded into nanofibers. The CNFs’ diameter ranges between 90 and 120 nm. In the MnTi/CNF samples, the agglomerates display widely varying sizes, as also suggested by the granulometric study based on the SEM data (mean particle size: 0.57(0.54) μm).

**Figure 5:**
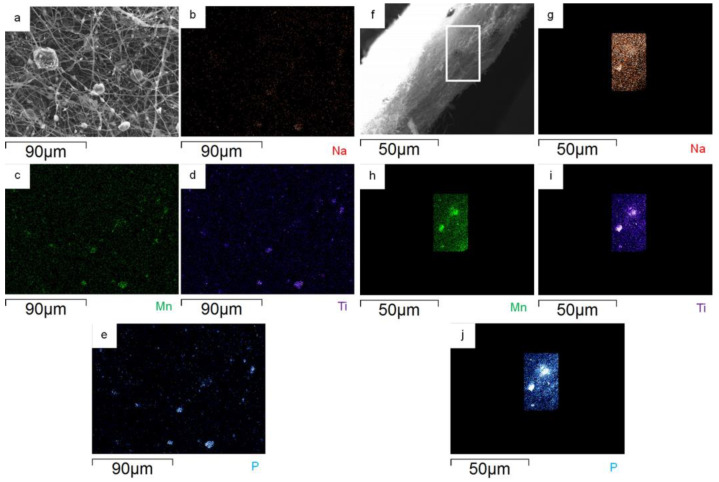
On the left surface: portion of 10%MnTi/CNF sample (**a**) and distribution maps of Na (**b**), Mn (**c**), Ti (**d**), and P (**e**). On the right: cross-section portion of 10%MnTi/CNF sample (**f**) and distribution maps of Na (**g**), Mn (**h**), Ti (**i**), and P (**j**).

**Figure 6:**
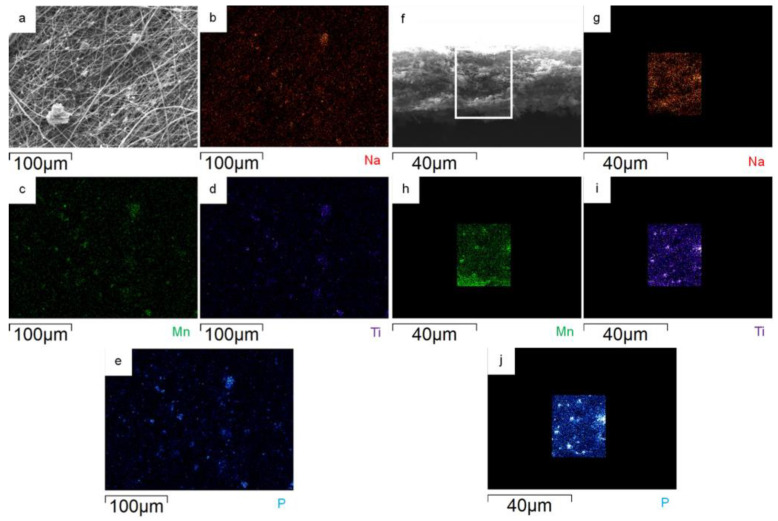
On the left: surface portion of 30%MnTi/CNF sample (**a**) and distribution maps of Na (**b**), Mn (**c**), Ti (**d**), and P (**e**). On the right: cross-section portion of 30%MnTi/CNF sample (**f**) and distribution maps of Na (**g**), Mn (**h**), Ti (**i**), and P (**j**).

The surface and bulk distribution of MnTi powder in the MnTi/CNF self-standing electrodes is investigated by EDS. The Na, Mn, Ti, and P distribution maps on the surface of the 10%MnTi/CNF sample (Figure 5a–e) confirm that the active material aggregates within and between CNFs and that it is homogeneously spread in CNFs. What is noteworthy is that the particles of the active material are spread along the sheet thickness, as demonstrated by the cross-section element distribution maps (Figure 5f–l); this is beneficial to obtain enhanced electrochemical performances, and it is not easily achieved by the dip- and drop-coating loading approach. Comparable EDS results are obtained for 30%MnTi/CNF, as demonstrated by the element distribution maps on the surface (Figure 6a–e) and cross-section (Figure 6f–l).

### 3.3. Thermogravimetric Analysis

**Figure 7:**
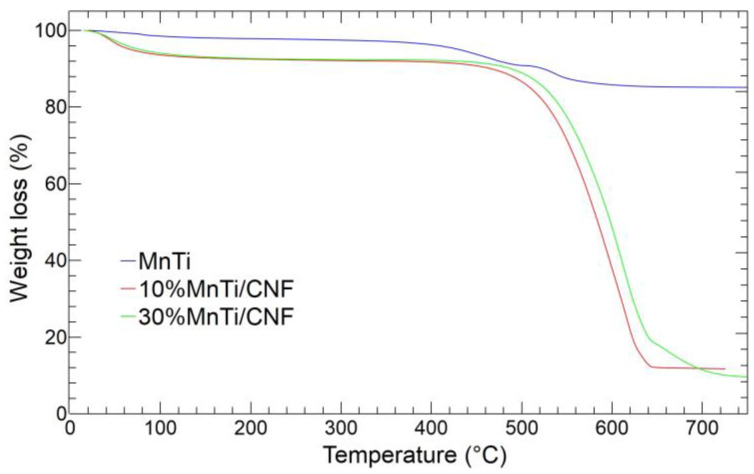
TGA curves of MnTi (blue), 10%MnTi/CNF (red), and 30%MnTi/CNF (green) samples. The analysis is performed in air between 25 and 730 °C.

The carbon content and the Na_3_MnTi(PO_4_)_3_ amount loaded into CNFs is evaluated by thermogravimetric analysis on the samples after the carbonization process. The thermogravimetric curves of MnTi, 10%MnTi/CNF, and 30%MnTi/CNF samples are shown in Figure 7.

The MnTi TGA curve displays three weight losses: 1.43 wt% at 100 °C, 13.88 wt% at 400–500 °C, and 0.89 wt% at 600 °C. The first loss is due to the release of adsorbed water, and the second and third account for the carbon coating content in the MnTi sample: 14.77 wt%. Finally, the small weight increase at 500 °C is explained by the oxidation of the low-valence state of metal species induced by carbon combustion, as reported in the literature [40,53].

The TGA curves of the 10%MnTi/CNF (red) and 30%MnTi/CNF (green) samples also show two mass losses: (i) below 100 °C due to the adsorbed water release (about 7.7 wt% for both samples); (ii) in the 500–650 °C temperature range, involving the carbonaceous component combustion of both the Na_3_MnTi(PO_4_)_3_ carbon coating and the carbon nanofibers. The latter mass loss corresponds to the carbon content in the samples: 80.57 wt% for 10%MnTi/CNF and 82.71 wt% for 30%MnTi/CNF. The 10%MnTi/CNF and 30%MnTi/CNF samples give a residual mass of 11.73 wt% at 650 °C and 9.59 wt% at 700 °C, respectively, which corresponds to the active material amount in the samples. The result matches the synthesis value of the 10%MnTi/CNF self-standing electrode. In the case of the 30%MnTi/CNF sample, the Na_3_MnTi(PO_4_)_3_ amount is much lower than the synthesis value (30 wt%). The TGA results match the values of the degree of crystallinity obtained by XRPD analysis (Table S1). We repeated the synthesis of the 30%MnTi/CNF sample, and we noticed that some sedimentation of the active material occurred along the tube connecting the pump and the needle of the horizontal spinneret. We believed this may have been due to the possible poor dispersibility of the pre-synthesized active material aggregates in the PAN-based polymer solution. We also tried to prepare the self-standing electrode using vertical equipment, but again, the Na_3_MnTi(PO_4_)_3_ 30 wt% amount was not achieved. Based on the above-mentioned results, we decided to investigate the electrochemical performances of the MnTi (tape-casted) and 10%MnTi/CNF (self-standing) electrodes.

### 3.4. Raman Analysis

The Raman analysis is performed to obtain insights on the structural compositions of the investigated materials. In particular, the aim is to study the Raman activity of carbon modes in both carbon coatings and CNFs, thus determining the order degree of the carbon component itself.

**Figure 8:**
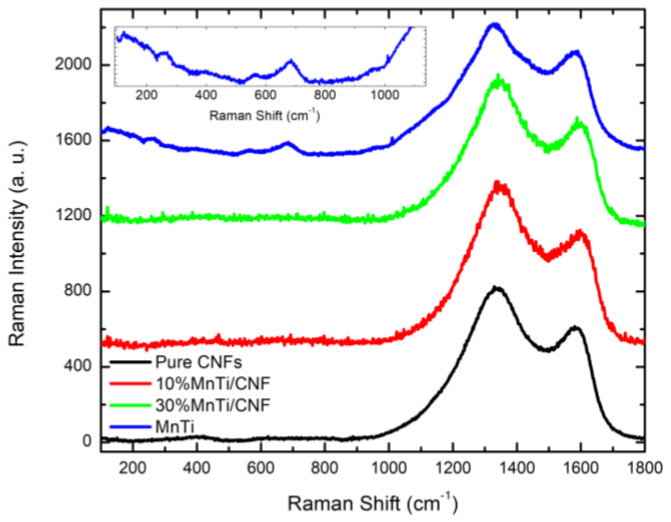
Room temperature Raman spectra of pure CNFs, 10%MnTi/CNF, 30%MnTi/CNF, and MnTi powder (from bottom to top).

The room temperature spectra of the 10%MnTi/CNF and 30%MnTi/CNF self-standing samples together with the ones of the MnTi powder and the only CNFs are reported in Figure 8.

The MnTi spectrum displays some broad signals in the low-frequency region (see inset). According to [56], the most prominent features at about 565 and 680 cm^−1^ could be ascribed to P–O and P–O–Na vibrations together with possible contribution by Ti–O ones, while less intense modes are visible below 300 cm^−1^ and they could arise from the vibrations involving Mn ions.

Apart from the MnTi sample that shows low-energy modes, each spectrum is dominated only by two intense modes that fall at about 1340 and 1585 cm^−1^. These features are a characteristic signature of carbonaceous materials, through which it is possible to obtain information about the order/disorder as well as the crystalline quality of the structure. Indeed, as it is well known, the mode occurring at about 1585 cm^−1^ is the G band and it is characteristic of an ordered graphitic network, while the one at ≈1340 cm^−1^ is known as the D band and it is generally ascribed to defectiveness [57].

The presence of the D and G bands also in the MnTi spectrum confirms the presence of a carbon coating on the MnTi particles, as evidenced by the morphological analysis and TGA data. The D mode seems to be broader than in the other samples, pointing towards a more disordered structure, consistent with an external citric carbon coating.

**Figure 9:**
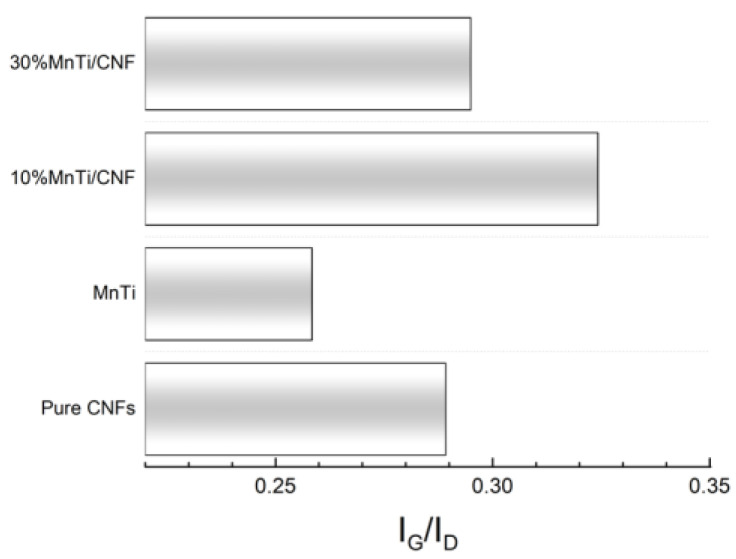
I_G_/I_D_ values reported for each investigated sample.

In Figure 9, the value of the I_G_/I_D_ ratio is reported for each sample. In general, the I_G_ and I_D_ values correspond to the intensities of the modes. However, to better account for the broadening of the peak, especially in the case of the MnTi sample, we have used the integrated intensity of the two Gaussian functions used to perform the best fitting procedure.

The smallest I_G_/I_D_ value (0.26) is obtained for the MnTi sample as expected: CNFs are the main source of ordered carbon, beneficial for the electronic conductivity enhancement of the electrodes. What is noteworthy is that a higher I_G_/I_D_ value is detected in the 10%MnTi/CNF sample, which will be electrochemically evaluated. This fact could be due to a beneficial effect of the native carbon coating of MnTi particles which favors an ordered carbon nanofiber adhesion. This effect should be more evident for low amounts of MnTi aggregates due to the weight of different Raman yields in the whole Raman spectrum of the sampled volumes.

### 3.5. Electrochemical Characterization

The Na_3_MnTi(PO_4_)_3_ shows three redox peaks of Ti^3+^/Ti^4+^, Mn^2+^/Mn^3+^, and Mn^3+^/Mn^4+^ at 2.1 V, 3.5 V, and 4.0 V, respectively. The presence of three different redox reactions makes the material suitable for different applications that require different applied voltages: as an anode for the Ti^3+^/Ti^4+^ redox couple and as a cathode thanks to Mn^2+^/Mn^3+^ and Mn^3+^/Mn^4+^ ones [53].

The redox mechanism involves a multielectron process during the Na^+^ extraction/insertion. It includes a two-electron transfer for Mn^2+^/Mn^3+^ and Mn^3+^/Mn^4+^ and one electron process in the case of Ti^3+^/Ti^4+^; an ex situ X-ray diffraction investigation demonstrates the sodiation/desodiation process involves both solid–solution and two-phase reactions [41,53]. Equations (1) and (2) summarize the charge/discharge process [41,53]:

(1) $$\mathrm{discharge}:{\;\mathrm{Na}}_{1}\mathrm{MnTi}{\left({\mathrm{PO}}_{4}\right)}_{3}\underset{4.0\;V\;}{\to }{\;\mathrm{Na}}_{2}\mathrm{MnTi}{\left({\mathrm{PO}}_{4}\right)}_{3}\underset{3.5\;V\;}{\to }{\;\mathrm{Na}}_{3}\mathrm{MnTi}{\left({\mathrm{PO}}_{4}\right)}_{3}\;\underset{2.1\;V}{\to }{\;\mathrm{Na}}_{4}\mathrm{MnTi}{\left({\mathrm{PO}}_{4}\right)}_{3}$$

(2) $$\mathrm{charge}:{\;\mathrm{Na}}_{4}\mathrm{MnTi}{\left({\mathrm{PO}}_{4}\right)}_{3}\underset{2.1\;V}{\to }{\;\mathrm{Na}}_{3}\mathrm{MnTi}{\left({\mathrm{PO}}_{4}\right)}_{3}\underset{3.5\;V}{\to }{\;\mathrm{Na}}_{2}\mathrm{MnTi}{\left({\mathrm{PO}}_{4}\right)}_{3}\;\underset{4.0\;V}{\to }{\;\mathrm{Na}}_{1}\mathrm{MnTi}{\left({\mathrm{PO}}_{4}\right)}_{3}$$

The Na_2_MnTi(PO_4_)_3/_Na_1_MnTi(PO_4_)_3_ process is kinetically favored with the fastest Na^+^ diffusion, as also supported by density functional theory calculations [53].

#### 3.5.1. Cyclic Voltammetry

**Figure 10:**
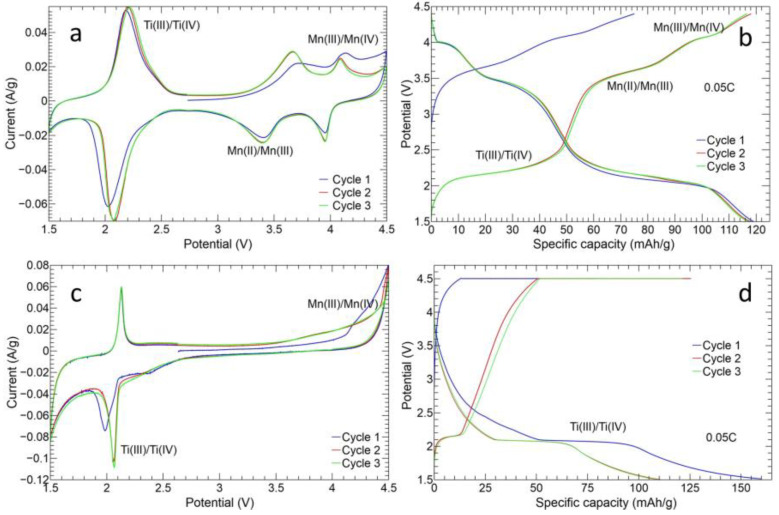
Cyclic voltammetry curves and first three charge/discharge profiles of MnTi tape-casted (**a**,**b**) and 10%MnTi/CNF (**c**,**d**) electrodes. The analysis is performed at 0.1 mV/s in the 1.5–4.5 V range.

The MnTi CV curve is shown in Figure 10a. The tape-casted electrode displays the three redox peaks of Ti^3+^/Ti^4+^, Mn^2+^/Mn^3+^, and Mn^3+^/Mn^4+^ at 2.20 V/2.06 V, 3.67 V/3.41 V, and 4.08 V/3.95 V, respectively. The ΔV of Ti^3+^/Ti^4+^ peak is 139 mV and progressively diminishes for Mn^2+^/Mn^3+^ (26 mV) and Mn^3+^/Mn^4+^ (13 mV). It indicates a quite small polarization phenomenon. The current intensity is higher than 0.04 A/g and lower than −0.06 A/g for anodic and cathodic peaks, respectively. The three redox couples are also consistent with the three plateaus detected in the Galvanostatic charge–discharge profiles shown in Figure 10b. In the first charge process, the plateau of the Ti^3+^/Ti^4+^ redox couple is not observed due to the initial open-circuit voltage (2.5–2.7 V). The CV curves and charge/discharge profiles of the 10%MnTi/CNF sample are reported in Figure 10c and Figure 10d, respectively. In this case, only the Ti^3+^/Ti^4+^ redox peaks at 2.12 V/2.06 V are detected, while the Mn^2+^/Mn^3+^ and Mn^3+^/Mn^4+^ ones become very faint. The sluggish redox activity may be attributed to the cell shrinkage at a sintering temperature of 750 °C [55] and to the intrinsic anti-site defect: Mn occupies the Na(2) vacancy and hampers the sodium ion diffusion and the manganese redox processes [39]. However, a temperature of 750 °C is necessary to obtain a successful carbonization. Secondly, we underline that the 10%MnTi/CNF electrode contains only 10 wt% of active material against the 70 wt% present in the MnTi tape-casted electrode, and this can influence the CV. The Ti^3+^/Ti^4+^ redox reaction is also confirmed by the evident plateau in Figure 10d. In the CV curves (Figure 10c), the weak peak at 2.4 V is explained by the disproportion reaction of Mn^3+^ dissolved in the electrolyte [41]. Finally, the cycles are overlapped to demonstrate a strong redox reversibility.

**Figure 11:**
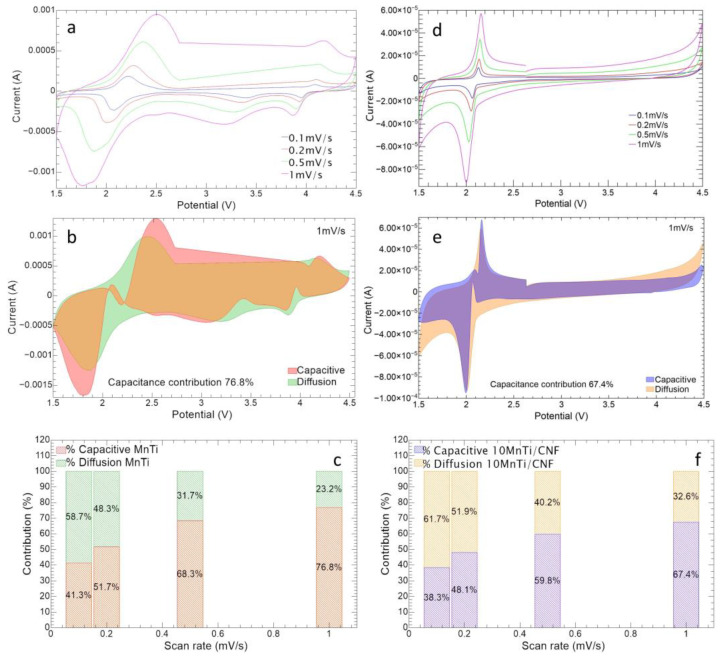
Cyclic voltammetry at different scan rates for MnTi tape-casted (**a**) and 10%MnTi/CNF (**d**) electrodes. Capacitive and diffusive contributions at 1 mV/s for MnTi tape-casted (capacitive: red; diffusive: green; both contributions: light brown) (**b**) and 10%MnTi/CNF (capacitive: blue; diffusive: orange; both contributions: violet) (**e**) electrodes. Capacitance and diffusion histogram for MnTi tape-casted (**c**) and 10%MnTi/CNF (**f**).

Figure 11a,d show the CV curves at different scan rates for MnTi and 10%MnTi/CNF samples, respectively. The data were analyzed to evaluate the sodium ion diffusion coefficient, the alkali metal-ion faradaic contribution (diffusion control), and the non-faradaic one (pseudo-capacitance control) caused by the formation of the double layer at the material surface [41,53,55]. The pseudo-capacitance contributions are shown in Figure 11b,c,e,f for the MnTi and 10%MnTi/CNF samples, respectively. The relationship between the redox current i_p_ (A) and scan rate *ν* (mV/s) is given by the following:

(3) $$i_{p}=k_{1}\nu +k_{2}\sqrt{\nu }$$

where i_p_ is the peak current, *ν* is the scan rate, and k_1_ and k_2_ are adjustable parameters [41,53]. The diffusion contribution is driven by the square root term thanks to the derivation of the i_p_ by the Randles–Sevick equation:

(4) $$i_{p}=2.69\times 10^{5}n^{\frac{3}{2}}\mathrm{AC}\sqrt{D\nu }$$

where n is the number of electrons transferred, A is the electrode area, C is the Na^+^ concentration and D is the diffusion coefficient. The quantities are given in the CGS unit system and at standard conditions. From Equations (3) and (4), the diffusion coefficient D can be evaluated by calculating the k_2_ term for both MnTi and 10%MnTi/CNF samples.

The tape-casted electrode (A = 0.78 cm^2^) exhibits diffusion coefficients values for anodic peaks of D_Ti(III)/Ti(IV)_ = 1.3 × 10^−9^ cm^2^/s, D_Mn(II)/Mn(III)_ = 8.4 × 10^−9^ cm^2^/s, and D_Mn(III)/Mn(IV)_ = 1.6 × 10^−10^ cm^2^/s. In the case of the cathodic peak, the D values are D_Ti(III)/Ti(IV)_ = 2.2 × 10^−9^ cm^2^/s, D_Mn(II)/Mn(III)_ = 7.6 × 10^−9^ cm^2^/s, and D_Mn(III)/Mn(IV)_ = 1.4 × 10^−9^ cm^2^/s. The results compare to the literature ones [53,55].

An equivalent analysis can be conducted for the self-standing electrode as well. Contrary to the MnTi, the 10%MnTi/CNF electrode features a three-dimensional structure, whose main advantage is the substantial increase in the active material surface. Indeed, 10%MnTi/CNF area A cannot be estimated from the electrode section. Therefore, we evaluate an equivalent anodic diffusion coefficient D_eq_ = 2.6 × 10^−12^ cm^2^/s and cathodic D_eq_ = 1.0 × 10^−11^ cm^2^/s for Ti^3+^/Ti^4+^ redox couple, where we assume electrode area A is equal to its section. The result is consistent with the fact that the Ti^3+^/Ti^4+^ self-standing electrode shows a lower current intensity than its tape-casted counterpart (see Figure 11a,d), and this depends on the Na_3_MnTi(PO_4_)_3_ amount in the 10%MnTi/CNF and MnTi electrodes (10 and 70 wt%, respectively). The low amount of the active material leads to a low electrochemical active area.

To compare the performance of tape-casted and self-standing electrodes, the capacity contribution of diffusion and reaction (capacitive contribution) control as a function of CV sweep rates were calculated. As shown in Figure 11e,f, the 10%MnTi/CNF sample exhibits a higher contribution of diffusion control at each scan rate than its tape-casted counterpart (Figure 11b,c), thanks to the presence of the very porous CNF sheets. Indeed, the porosity of non-woven nanofibers guarantees an easier electrolyte diffusion which easily makes contact with active material particles, as reported in the literature [58,59]. This is also confirmed by the complete electrolyte permeation of the 10%MnTi/CNF sheet after carbonization, as shown in Figure S3 (Supporting Information). Finally, the tape-casted anodic and cathodic peaks tend to move to the right and left, respectively as the scan rate increases (Figure 11a), while this behavior is less pronounced for the self-standing sample (Figure 11d). For example, the Ti^3+^/Ti^4+^ redox peak overpotentials of the MnTi sample are 139 mV, 280 mV, 503 mV, and 768 mV as the scan rate increases against 63 mV, 58 mV, 119 mV, and 154 mV for the 10%MnTi/CNF. The results suggest more irreversible redox processes in the tape-casted electrode than in the self-standing one.

#### 3.5.2. Charge/Discharge Cycles

**Figure 12:**
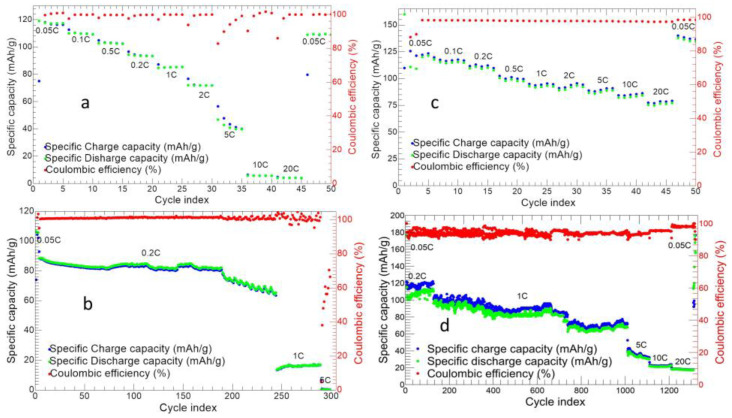
Charge/discharge cycling at different C-rate of MnTi tape-casted (**a**) and 10%MnTi/CNF (**c**) electrodes. Long charge/discharge cycles for MnTi tape-casted (**b**) and 10%MnTi/CNF (**d**) electrodes.

Figure 12 shows the charge/discharge analysis for both MnTi and 10%MnTi/CNF samples.

The MnTi displays an initial charge and discharge capacity of 75.02 and 119.08 mAh/g, respectively (Figure 12a). The initial charge capacity is lower than the following cycles because the OCV (between 2.5–2.8 V) is higher than the Ti^3+^/Ti^4+^ redox potential. We obtain average discharge capacities of 117.76, 109.80, 102.69, 93.67, 85.09, 71.83, 42.03, 5.70, and 4.12 mAh/g at 0.05C, 0.1C, 0.2C, 0.5C, 1C, 2C, 5C, 10C, and 20C, respectively. The cell exhibits a good capacity recovery at the end of the measurement and a coulombic efficiency ≥98%. In Figure 12b, the long charge/discharge cycles are shown. After the first five cycles at 0.05C, the cell is tested at 0.2C, 1C, and 5C for 240, 50, and 20 cycles, respectively. The initial discharge capacity is 106.48 mAh/g, and the cell exhibits a coulombic efficiency ≥99%. The specific capacity decreases, increasing both the cycle index and C-rate. For the first 190 cycles at 0.2C, the average capacity value is 84.24 mAh/g, while it diminishes to 71.63 mAh/g in the following 50 ones at the same C-rate with a capacity retention of 73.97% at the 240th cycle. By increasing the C-rate from 0.2C to 1C, the capacity value decreases to 16.18 mAh/g, while at 5C, the cell does not work. So, it can be concluded that the MnTi sample does not support C-rates higher than 1C after 290 cycles.

The 10%MnTi/CNF shows an initial charge and discharge capacity of 109.9 and 160.04 mAh/g, respectively (see Figure 12c). We obtain average discharge capacities of 124.38, 115.68, 111.04, 100.68, 93.6, 91.42, 89.15, 88.30, and 77.60 mAh/g at 0.05C, 0.1C, 0.2C, 0.5C, 1C, 2C, 5C, 10C, and 20C, respectively. At the end of the measurement, the cell completely recovers the initial capacity with a coulombic efficiency ≥98%. Contrary to the tape-casted MnTi electrode, the self-standing 10%MnTi/CNF one exhibits (i) a very moderate capacity loss by increasing the C-rate, (ii) good stability and reversibility of sodium storage, and (iii) promising performances also at C-rates higher than 5C. The 10%MnTi/CNF ability to cycle at high C-rates is guaranteed by the CNFs 3D network that gives high porosity and an easier diffusion of Na-ion. This implies a high power density, as reported by Vu and co-workers [58]. The capacity values of the 10%MnTi/CNF electrode shown in Figure 12c are comparable to the literature ones [40,41]. Notably, these promising electrochemical performances are obtained on an electrode synthesized by a simple and feasible approach with an active material amount of 10 wt%, against the 70–80 wt% of the conventional tape-casted electrodes. We underline some drawbacks can be envisaged in the sintering temperature of 750 °C necessary for the CNFs’ graphitization as this implies cell shrinkage and the sluggishness of the redox process. Nevertheless, they are overcome thanks to the advantages of the CNFs: they provide conducive carbon, a porous matrix beneficial for electrolyte–active material contact, easy sodium ions diffusion, and a light and self-standing electrode.

The very promising electrochemical performances of the 10%MnTi/CNF sample are also confirmed by the long-term charge/discharge cycling shown in Figure 12d: the cell lifespan is tested at 0,05C, 0.2C, 1C, 5C, 10C, and 20C for 5, 50, 1000, 100, 100, and 100 cycles, respectively. At 0.05C, the discharge capacity of 173.4 mAh/g in the first cycle approaches the theoretical one, and then values of about 120 mAh/g are achieved. The specific capacity decreases, increasing the cycle index and at the C-rate change. The specific capacity ranges between 67.3 mAh/g and 110.1 mAh/g for 1000 cycles at 1C. Notably, after 1000 cycles at 1C, the cell can be further cycled at higher C-rates with a final capacity retention of 59.6%. Contrary to the tape-casted electrode for which the capacity dramatically decreases at 1C (16.18 mAh/g) after 250 cycles at 0.2C (Figure 12b), the self-standing 10%MnTi/CNF electrode shows a longer lifespan, and its capacity is completely recovered in the last ten cycles at 0.05C after 1350 cycles. The good long-term cycling is another advantage of using CNFs for the electrode. As reported by the Vu group [58], the porosity facilitates the diffusion of the ions in bulk electrodes and also buffers the volume change during the charge and discharge cycle, providing a longer cell lifespan. The coulombic efficiency is ≥93% which implies a lower redox reversibility, especially in the first 50 cycles. Upon increasing the C-rate, the specific capacity is 33.6, 22.6, and 17.3 mAh/g at 5C, 10C, and 20C, respectively. Finally, we performed electrochemical impedance spectroscopy measurements on the cycled tape-casted and 10%MnTi/CNF self-standing electrodes. The Nyquist plot is shown in Figure S4. The impedance spectra compare to the literature ones for the Na_3_MnTi(PO_4_)_3_ active material with different carbon coatings (with and without graphene oxide) [40]. The smaller diameter of the semicircle in the high-frequency region for the 10%MnTi/CNF electrode confirms smaller charge transfer resistance (585.7 Ω vs. 803.8 Ω of the tape-casted cathode) and a faster charge transfer at the electrode–electrolyte interface. The larger slope of the Warburg impedance of the 10%MnTi/CNF indicates more favorable Na ion transport in the self-standing electrode compared to the tape-casted one.

## 4. Conclusions

In this study, a simple and viable approach is used to synthesize self-standing electrodes for SIBs based on Na_3_MnTi(PO_4_)_3_ active material loaded into carbon nanofibers by electrospinning. The CNFs demonstrate to be a suitable matrix to host Na_3_MnTi(PO_4_)_3_ particles. The active material is detected both in and within CNFs and it is homogeneously distributed along the sheet thickness. The loaded Na_3_MnTi(PO_4_)_3_ maintains its NASICON-type crystal structure, but the sintering temperature of 750 °C used for the carbonization process induces cell shrinkage. While this implies a sluggish redox activity, the presence of the very porous non-woven nanofibers guarantees an easier electrolyte diffusion and an increased alkali metal-ion faradaic contribution. The charge/discharge cycling tests at different C-rates and long-term cycling investigations confirm the promising electrochemical performances of the self-standing electrode compared to its conventional tape-casted counterpart. The 10%MnTi/CNF electrode displays an initial discharge capacity of 124.38 mAh/g at 0.05C, which is completely recovered at the end of the measurement with a coulombic efficiency ≥98%. The capacity value at 20C is 77.60 mAh/g. The self-standing electrode gives an improved lifespan compared to the tape-casted one: it exhibits capacities in the 67.2–110.1 mAh/g range at 1C and can further be cycled at 5C, 10C, and 20C after 1000 cycles at 1C (total cycles: 1350), contrary to the tape-casted one working only for 300 cycles and up to 5C. Notably, the enhanced capacity and cycling performances are obtained by only 10 wt% of active material loading into CNFs compared to 70 wt% of its tape-casted counterpart.

## Data Availability

The data presented in this study are available on request from the corresponding author.

## Supplementary Materials

The following supporting information can be downloaded at: https://www.mdpi.com/article/10.3390/nano14090804/s1, Figure S1: Rietveld refinement of the X-ray diffraction data of the Na3MnTi(PO4)3 pristine material and CNF-loaded Na3MnTi(PO4)3 samples; Figure S2: SEM images of the electrospun and graphitized 10%MnTi/CNF sample; Figure S3: electrolyte permeation in 10%MnTi/CNF sample; Figure S4: Nyquist plot of the 10%MnTi/CNF and MnTi electrodes; Table S1: Lattice parameters, cell volume, *c/a* ratio, crystallite size, crystallinity percentage, and discrepancy factors obtained by the Rietveld refinement of the diffraction data of the pristine and Na_3_MnTi(PO_4_)_3_/CNF samples.
