# Supplementary material for: Na3MnTi(PO4)3/C Nanofiber Free-Standing Electrode for Long-Cycling-Life Sodium-Ion Batteries
Source: Nanomaterials (Basel). 2024 May 5;14(9):804. doi: 10.3390/nano14090804 (PMC11085064; doi:10.3390/nano14090804)
Supplement: Supplementary file 1 [file nanomaterials-14-00804-s001.zip › nanomaterials-2980552-supplementary.pdf]

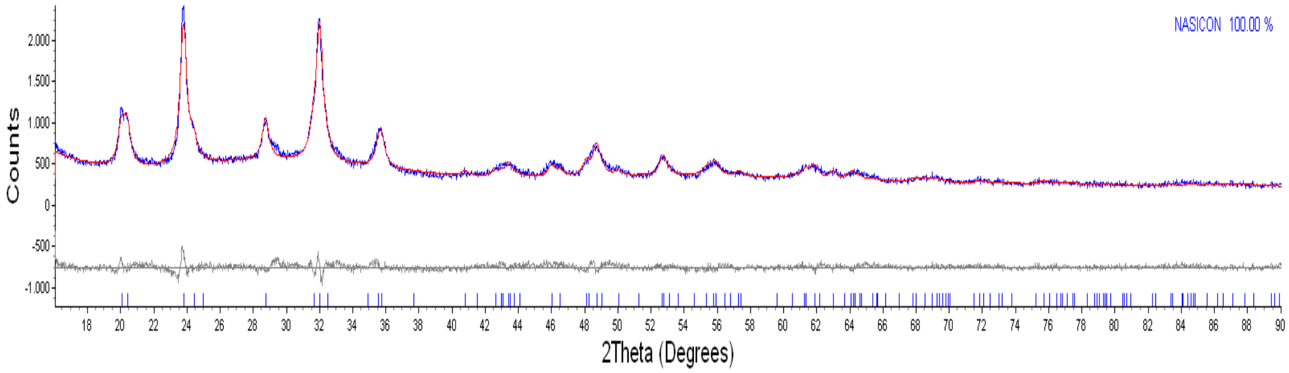

(a)

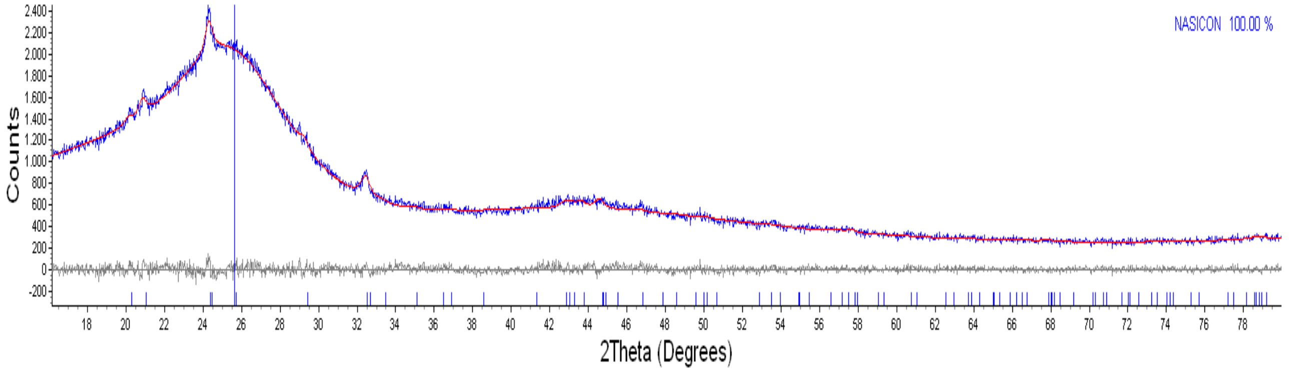

(b)

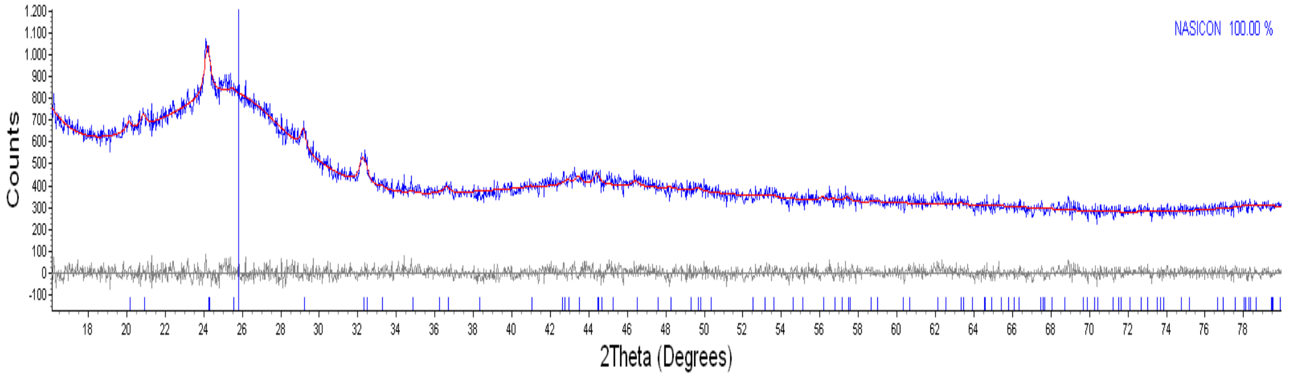

(c)

Figure S1. Rietveld refinement of the X-ray diffraction data of the (a) MnTi, (b) 10%MnTi/CNF and (c) 30%MnTi/CNF samples. Experimental pattern (blue line), calculated pattern (red line), difference curve (grey line). Peaks position of the $Na_3MnTi(PO_4)_3$ phase (blue bars).

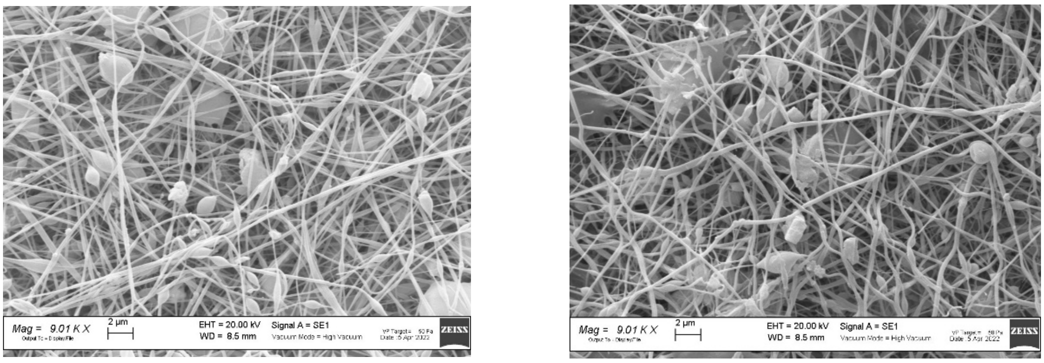

(a) (b)

Figure S2. SEM images of (a) electrospun and (b) carbonized 10%MnTi/CNF sample.

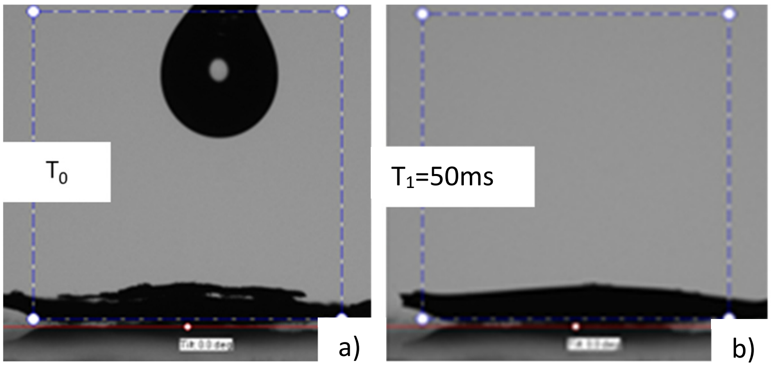

Figure S3. The electrolyte permeation in 10%MnTi/CNF after carbonization.

As shown in Figure S3, the electrolyte permeation is complete and fast in 10%MnTi/CNF carbonized sample. After 50 ms the electrolyte is completely absorbed by the 3D CNFs network. The impregnation of the self-standing electrode is demonstrated, because the electrode swells. The contact angle cannot be evaluated due to the fast electrolyte adsorption.

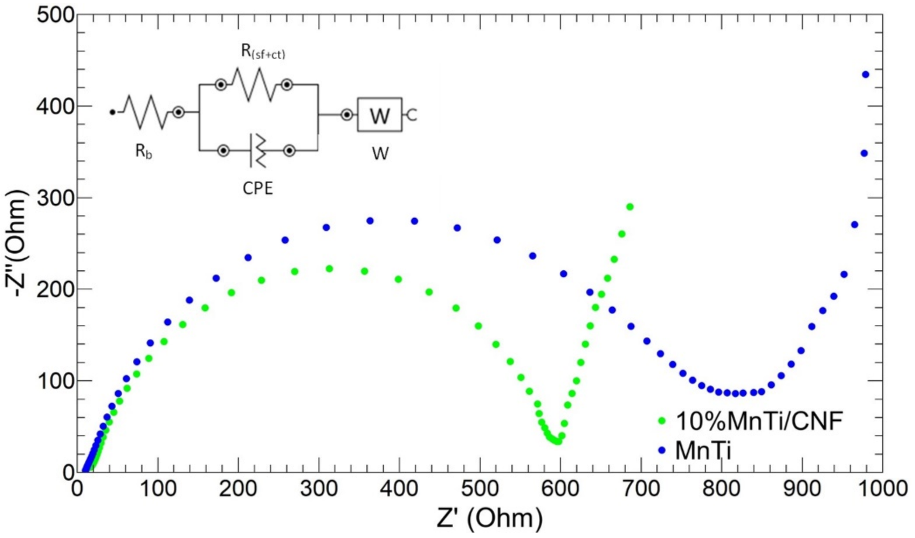

Figure S4. Nyquist plot of the 10%MnTi/CNF and MnTi electrodes. The equivalent circuit is shown in the inset. R_b: electrolyte resistance; $R_{(sf+ct)}$: surface and charge transfer resistance; W: Warburg impedance.

Table S1. $Na_3MnTi(PO_4)_3$ lattice parameters, crystallite size, degree of crystallinity, discrepancy factor and goodness of fit obtained by the Rietveld refinement of the MnTi and MnTi/CNF samples. Cell volume and *c/a* ratio is also reported.

**SAMPLE**	**MnTi**	**10%MnTi/CNF***	**30%MnTi/CNF***
a (Å)	8.696(2)	8.441(6)	8.462(8)
c (Å)	21.851(5)	21.834(19)	21.911(29)
V (Å^3)	1431.00	1347.26	1358.75
c/a	2.51	2.59	2.59
Crystallite size (nm)	18.7(3)	19.0(1.4)	25.2(2.3)
Crystallinity (%)	-	9.25	8.1
R_{wp}	6.26	4.20	5.13
S	1.34	1.10	1.07

* Refined parameters:

(i) global parameters: polynomial background (order 2) and sample displacement;

(ii) $Na_3MnTi(PO_4)_3$ phase: scale factor, crystallite size (Profile model: Fundamental Parameters approach), lattice parameters;

(iii) amorphous broad band: 2θ position, intensity, and peak broadening.
